# A Cluster Analysis of Oral and Cognitive Health Indicators in the CLSA: An Exploratory Study on Cholinergic Activity as the Link

**DOI:** 10.1177/23800844231190834

**Published:** 2023-08-22

**Authors:** K. Rohani, B. Nicolau, S. Madathil, L. Booij, D. Jafarpour, P.B. Haricharan, J. Feine, R. Alchini, F. Tamimi, R. de Souza

**Affiliations:** 1 Faculty of Dental Medicine and Oral Health Sciences, McGill University, Montreal, Canada; 2 Psychology Department, Concordia University, Montreal, Canada; 3 College of Dental Medicine, Qatar University, Doha, Qatar

**Keywords:** aging, bone density, cholinergic neurons, CLSA, cognition, mouth diseases, oral health

## Abstract

**Introduction:**

Poor oral health has been suggested as a risk factor for cognitive decline. Yet, biologically plausible mechanisms explaining this relationship remain unknown.

**Objectives:**

We aimed (1) to identify oral and cognitive health clustering patterns among middle-aged to elderly Canadians and (2) to investigate the extent to which these patterns could be explained by bone mineral density (BMD), a proxy measure of the cholinergic neurons’ activity.

**Methods:**

This cross-sectional study used baseline data from the Comprehensive cohort of the Canadian Longitudinal Study of Aging (CLSA). Oral health was assessed by a self-report questionnaire, and 7 task-based instruments measured cognitive health. We identified oral and cognitive health clusters, our outcome variables, using latent class analysis. Two sets of multivariate logistic regression and 95% confidence intervals were used to investigate whether BMD explains the odds of membership in a certain oral and cognitive health group. The final models were adjusted for socioeconomic, health, and lifestyle factors.

**Results:**

Our study sample (N = 25,444: 13,035 males, 12,409 females) was grouped into 5 and 4 clusters based on the oral health status and performance on the cognitive tasks, respectively. After adjusting for all potential covariates, increase in BMD was not associated with higher odds of membership in classes with better oral health (odds ratio [OR] = 1.58 [95% confidence interval {CI}: 0.85–2.92]) and cognitive health (OR = 1.61 [95% CI: 1–2.6]) compared with the groups with the least favorable oral and cognitive health status, respectively.

**Conclusion:**

Middle-aged and elderly Canadians show different oral and cognitive health profiles, based on their denture-wearing status and performance on cognitive tests. No evidence could be found to support BMD in place of cholinergic neurons’ activity as the common explanatory factor behind the association between oral health and cognitive health.

**Knowledge Transfer Statement:**

This study is probably the first of its kind to shed light on the cholinergic system as a potential pathway influencing oral and cognitive health. Our findings may support the notion that any potential association between poor oral health and cognitive health might be explained by common contributors, helping clinicians to find the common risk factors for both conditions.

## Introduction

Oral diseases are among the most common health conditions, affecting more than 3.5 billion people globally (Kassebaum et al. 2017). Not only can oral diseases cause public health burden (Sheiham et al. 2015), but they also are known to induce chronic health conditions (Nakamura et al. 2021). Recent studies have proposed that poor oral health could be a risk factor for cognitive decline, and specifically, Alzheimer’s disease (AD; Leira et al. 2017). Several mechanisms have been put forth to explain the biological plausibility of this oral-systemic connection (Borsa et al. 2021). Among these are the chronic inflammation caused by periodontitis (Dioguardi et al. 2020), reduced sensory input resulting from ineffective mastication (Lopez-Chaichio et al. 2021), and nutritional deficiency attributable to masticatory dysfunction (Kim et al. 2017).

While there is a strong biological plausibility for these mechanisms, results from studies investigating them are far from conclusive (Tonsekar et al. 2017). In addition, they overlook the role of aging and age-related changes in cholinergic neurons’ activity affecting both oral and cognitive health. These neurons, which use acetylcholine, are widely spread throughout the body and are involved in distinct functions including learning (Deiana et al. 2011), memory (Robinson et al. 2011), saliva secretion (Baum 1993), and maintaining bone-mass density (Eimar et al. 2013). On one hand, the degeneration of these neurons results in memory deficit, leading to cognitive decline (Hasselmo and Sarter 2011). On the other hand, cholinergic system stimulation results in salivary secretion, the degeneration of which disrupts oral homeostasis (Dawes 2008).

Therefore, we hypothesized that an age-related degeneration of central and peripheral cholinergic neurons might be a common underlying contributor, explaining the deterioration of both cognitive and oral health. The cholinergic components are widely expressed in bone tissue, indicating their involvement in bone remodeling, with nicotinic and muscarinic receptors influencing bone turnover. In mice, stimulating nicotinic receptors results in greater bone mass by inducing osteoclast apoptosis, while stimulating muscarinic receptors in laboratory settings leads to enhanced osteoblast proliferation. Several studies have also reported an association between bone loss and clinical conditions related to reduced cholinergic activity (Eimar et al. 2013). Thus, in this cross-sectional study, we estimate the extent to which cholinergic system activity is independently associated with oral and cognitive health classifications. Bone mineral density (BMD) was the proxy measure of cholinergic neuron activity used in this study. Our research question was: To what extent is the clustering of study participants, based on their oral and cognitive health, explained by BMD as a proxy measure for cholinergic neurons’ function?

## Methods

We used baseline data from the Canadian Longitudinal Study of Aging (CLSA), which is a large, nationwide ongoing cohort study. A total of 51,338 Canadian men and women aged 45 to 85 y were randomly selected from 10 Canadian provinces and invited to participate.

CLSA participants comprised 2 main cohorts, namely, “Comprehensive” and “Tracking.” Compared with the Tracking cohort, the Comprehensive cohort underwent detailed physical examinations and in-person home interviews, including additional cognitive health assessments. Considering this, only baseline data obtained from this cohort, which consisted of 30,097 participants (Raina et al. 2009) were analyzed in this study. The sample for the comprehensive cohort was randomly chosen from eligible households in the provincial health care databases, who reside within 25- to 50-km distance from designated data collection centers. Persons living in the three territories, or federal First Nations reserves, or on other Indigenous settlements, people living in long-term care facilities, Canadian armed forces, and those with cognitive impairment at baseline were excluded (Raina et al. 2009).

This study was approved by the Institutional Review Board (IRB) of McGill University (IRB study number: A07-E51-18B), closely followed the “Sample Access Policy and Guiding Principles” specified by the CLSA team (Raina et al. 2009), and conformed to STROBE Guidelines.

### Measures

#### Indicators of oral health

The Comprehensive cohort provided information regarding their oral health status through “the maintaining contact questionnaire” administered by computer-assisted telephonic interviews. The oral health questionnaire was based on the Canadian Community Health Survey 2.1, incorporating subjective indicators of oral health status as proposed by Locker and Miller (1994). These self-reported indicators have shown acceptable test-retest reliability, internal consistency levels (>0.7), and concurrent and construct validity (Locker and Miller 1994). Questions assessed oral health status and service usage (Table 1).

**Table 1.** Oral Health Questions from the “Maintaining Contact Questionnaire” and Their Response Categories Included in the Latent Class Analysis Step.

	Question (Q)	Response Categories in the Final Data Set
1	In general, would you say the health of your mouth is excellent, very good, good, fair, or poor?	Excellent, Very Good, Good 1 Fair, Poor 2
2	Do you have one or more of your own original teeth?	Yes 1 No 2
3	Do you wear dentures or false teeth?	Yes 1 No 2
4	In the past 12 months, how often have you found it uncomfortable to eat any food because of problems with your mouth? Would you say . . .	Often, Sometimes 2 Rarely, Never 1
5	In the past 12 months, how often have you avoided eating particular foods because of problems with your mouth? Would you say . . .	Often, Sometimes 2 Rarely, Never 1
6	In the past 12 months have you experienced any of the following?	
	6.1. Toothache	Yes 2, No 1
	6.2. Cannot chew adequately	Yes 2, No 1
	6.3. Dentures uncomfortable	Yes 2, No 1
	6.4. Dentures loose/don’t fit	Yes 2, No 1
	6.5. Dentures broken	Yes 2, No 1
	6.6. Dentures missing	Yes 2, No 1
	6.7. Swelling in your mouth	Yes 2, No 1
	6.8. Dry mouth	Yes 2, No 1
	6.9. Burning mouth	Yes 2, No 1
	6.10. Jaw muscles sore	Yes 2, No 1
	6.11. Jaw joints painful	Yes 2, No 1
	6.12. Natural tooth decayed	Yes 2, No 1
	6.13. Natural tooth loose	Yes 2, No 1
	6.14. Natural tooth broken	Yes 2, No 1
	6.15. Gums around natural teeth are sore	Yes 2, No 1
	6.16. Gums around natural teeth bleed	Yes 2, No 1
	6.17. Denture-related sores	Yes 2, No 1
	6.18. Teeth or dentures dirty	Yes 2, No 1
	6.19. Bad breath	Yes 2, No 1

#### Indicators of cognitive health

Trained CLSA interviewers administered face-to-face cognitive tests, and participants’ responses were audio recorded. Subsequently, the CLSA personnel scored these tests using a standardized procedure (“CLSA Tracking and Comprehensive Cognition Measurements” 2019).

Cognitive functioning was assessed using 7 instruments in 3 different domains, namely, memory, executive functioning, and psychomotor speed (Raina et al. 2009). These 7 instruments are Rey Auditory Verbal Learning Test (RAVLT), Mental Alteration Test (MAT), (Teng 1995), Prospective Memory Test (PMT), Stroop Neuropsychological Screening Test (Troyer et al. 2006), Controlled Oral Word Association Test (COWAT), Animal Fluency Test (AFT), and Choice Reaction Time (CRT) (Tuokko et al. 2017). The CRT was not included in this study due to the roof effect, with respondents reaching an average score of 100%.

#### BMD

BMD was measured using Hologic Discovery ATM dual-energy X-ray absorptiometry, which is the gold standard method (Høiberg et al. 2016). BMD measures were recorded for both hips, lateral spine, forearm, and whole body. Our analysis used the whole-body BMD raw score as a proxy measure for the activity of cholinergic system.

#### Covariates

The selection of these covariates was guided by previous literature (Wu et al. 2016). These variables were divided into 3 categories: demographic and socioeconomic factors, chronic health conditions, and lifestyle factors (Tables 2 and 3).

**Table 2.** Descriptive Statistics of Selected Variables According to Oral Health Classes.

	Class 1, *n* = 15,922 (%)	Class 2, *n* = 822 (%)	Class 3, *n* = 1320 (%)	Class 4, *n* = 2251 (%)	Class 5, *n* = 5129 (%)	Overall, *N* = 25,444 (%)
Age (y)						
Mean (SD)	62.0 (9.96)	67.8 (9.49)	60.8 (9.44)	70.6 (8.76)	60.9 (9.63)	62.6 (10.1)
Median [Min, Max]	61.0 [45.0, 86.0]	68.0 [45.0, 85.0]	60.0 [45.0, 86.0]	71.0 [45.0, 86.0]	60.0 [45.0, 86.0]	62.0 [45.0, 86.0]
Sex						
Male	7971 (50.1)	441 (53.6)	780 (59.1)	1205 (53.5)	2638 (51.4)	13,035 (51.2)
Female	7951 (49.9)	381 (46.4)	540 (40.9)	1046 (46.5)	2491 (48.6)	12,409 (48.8)
Education						
>Secondary	527 (3.3)	131 (15.9)	71 (5.4)	370 (16.4)	177 (3.5)	1276 (5.0)
Secondary	1388 (8.7)	112 (13.6)	106 (8.0)	328 (14.6)	390 (7.6)	2324 (9.1)
Some post-secondary	1102 (6.9)	66 (8.0)	109 (8.3)	207 (9.2)	360 (7.0)	1844 (7.2)
Postsecondary	12,890 (81.0)	508 (61.8)	1030 (78.0)	1340 (59.5)	4195 (81.8)	19,963 (78.5)
Missing	15 (0.1)	5 (0.6)	4 (0.3)	6 (0.3)	7 (0.1)	37 (0.1)
Total household income						
<$20,000	419 (2.6)	130 (15.8)	130 (9.8)	251 (11.2)	272 (5.3)	1202 (4.7)
≥$20,000–<$50,000	2658 (16.7)	317 (38.6)	317 (24.0)	899 (39.9)	1022 (19.9)	5213 (20.5)
≥$50,000–<$100,000	5421 (34.0)	227 (27.6)	429 (32.5)	677 (30.1)	1749 (34.1)	8503 (33.4)
≥$100,000–$150,000	3379 (21.2)	68 (8.3)	212 (16.1)	172 (7.6)	978 (19.1)	4809 (18.9)
≥$150,000	3161 (19.9)	25 (3.0)	149 (11.3)	79 (3.5)	816 (15.9)	4230 (16.6)
Missing	884 (5.6)	55 (6.7)	83 (6.3)	173 (7.7)	292 (5.7)	1487 (5.8)
Ethnicity						
White	14,644 (92.0)	736 (89.5)	1158 (87.7)	2067 (91.8)	4609 (89.9)	23,214 (91.2)
Non-White	1278 (8.0)	86 (10.5)	162 (12.3)	184 (8.2)	520 (10.1)	2230 (8.8)
Diabetes						
Type I	69 (0.4)	6 (0.7)	8 (0.6)	19 (0.8)	33 (0.6)	135 (0.5)
Type II	1121 (7.0)	141 (17.2)	141 (10.7)	333 (14.8)	507 (9.9)	2243 (8.8)
Neither	14,652 (92.0)	664 (80.8)	1155 (87.5)	1858 (82.5)	4546 (88.6)	22,875 (89.9)
Missing	80 (0.5)	11 (1.3)	16 (1.2)	41 (1.8)	43 (0.8)	191 (0.8)
Hypertension						
Yes	6449 (40.5)	432 (52.6)	532 (40.3)	1312 (58.3)	2091 (40.8)	10,816 (42.5)
No	9166 (57.6)	358 (43.6)	754 (57.1)	858 (38.1)	2902 (56.6)	14,038 (55.2)
Missing	307 (1.9)	32 (3.9)	34 (2.6)	81 (3.6)	136 (2.7)	590 (2.3)
Alcohol consumption						
Regular drinker	12,488 (78.4)	515 (62.7)	858 (65.0)	1416 (62.9)	3781 (73.7)	19,058 (74.9)
Occasional drinker	1647 (10.3)	132 (16.1)	215 (16.3)	393 (17.5)	667 (13.0)	3054 (12.0)
No drinking	1475 (9.3)	154 (18.7)	204 (15.5)	368 (16.3)	583 (11.4)	2784 (10.9)
Missing	312 (2.0)	21 (2.6)	43 (3.3)	74 (3.3)	98 (1.9)	548 (2.2)
Smoking (categorical)						
Nonsmokers	8316 (52.2)	226 (27.5)	570 (43.2)	729 (32.4)	2373 (46.3)	12,214 (48.0)
Daily smokers	700 (4.4)	132 (16.1)	175 (13.3)	244 (10.8)	390 (7.6)	1641 (6.4)
Occasional smokers	241 (1.5)	10 (1.2)	15 (1.1)	27 (1.2)	101 (2.0)	394 (1.5)
Ex-smokers	5683 (35.7)	430 (52.3)	483 (36.6)	1157 (51.4)	1958 (38.2)	9711 (38.2)
Missing	982 (6.2)	24 (2.9)	77 (5.8%)	94 (4.2)	307 (6.0)	1484 (5.8)
Smoking (cigarette-years)						
Mean (SD)	28.7 (51.7)	82.9 (86.5)	46.8 (68.2)	74.1 (84.9)	36.5 (58.9)	37.0 (61.2)
Median [Min, Max]	0 [0, 490]	60.0 [0, 490]	6.00 [0, 384]	40.0 [0, 490]	0 [0, 392]	0 [0, 490]
Missing	1087 (6.8)	47 (5.7)	98 (7.4)	127 (5.6)	346 (6.7)	1705 (6.7)
Whole-body bone mineral density						
Mean (SD)	1.14 (0.13)	1.11 (0.14)	1.13 (0.13)	1.11 (0.14)	1.14 (0.13)	1.14 (0.13)
Median [Min, Max]	1.14 [0.71, 1.84]	1.10 [0.70, 1.75]	1.12 [0.77, 1.99]	1.10 [0.74, 1.75]	1.14 [0.74, 1.78]	1.13 [0.70, 1.99]
Missing	534 (3.4)	47 (5.7)	50 (3.8)	96 (4.3)	205 (4.0)	932 (3.7)

**Table 3.** Descriptive Statistics of Selected Variables According to Cognitive Status Class.

	Class 1, *n* = 12,173 (%)	Class 2, *n* = 4783 (%)	Class 3,*n* = 1989 (%)	Class 4, *n* = 6499 (%)	Overall, *N* = 25,444 (%)
Age (y)					
Mean (SD)	59.2 (8.80)	62.9 (9.57)	71.8 (9.34)	66.2 (10.1)	62.6 (10.1)
Median [Min, Max]	58.0 [45.0, 86.0]	62.0 [45.0,86.0]	74.0 [45.0, 86.0]	66.0 [45.0, 86.0]	62.0 [45.0, 86.0]
Sex					
Male	6290 (51.7)	2424 (50.7)	980 (49.3)	3341 (51.4)	13,035 (51.2)
Female	5883 (48.3)	2359 (49.3)	1009 (50.7)	3158 (48.6)	12,409 (48.8)
Education					
>Secondary	185 (1.5)	194 (4.1)	315 (15.8)	582 (9.0)	1276 (5.0)
Secondary	805 (6.6)	453 (9.5)	260 (13.1)	806 (12.4)	2324 (9.1)
Some postsecondary	807 (6.6)	360 (7.5)	182 (9.2)	495 (7.6)	1844 (7.2)
Postsecondary degree	10,368 (85.2)	3772 (78.9)	1224 (61.5)	4599 (70.8)	19,963 (78.5)
Missing	8 (0.1)	4 (0.1)	8 (0.4)	17 (0.3)	37 (0.1)
Income					
<$20,000	327 (2.7)	210 (4.4)	232 (11.7)	433 (6.7)	1202 (4.7)
≥$20,000–<$50,000	1680 (13.8)	987 (20.6)	719 (36.1)	1827 (28.1)	5213 (20.5)
≥$50,000–<$100,000	3943 (32.4)	1678 (35.1)	587 (29.5)	2295 (35.3)	8503 (33.4)
≥$100,000–<$150,000	2842 (23.3)	896 (18.7)	162 (8.1)	909 (14.0)	4809 (18.9)
≥$150,000	2834 (23.3)	727 (15.2)	87 (4.4)	582 (9.0)	4230 (16.6)
Missing	547 (4.5)	285 (6.0)	202 (10.2)	453 (7.0)	1487 (5.8)
Ethnicity					
White	11,265 (92.5)	4403 (92.1)	1771 (89.0)	5775 (88.9)	23,214 (91.2)
Non-White	908 (7.5)	380 (7.9)	218 (11.0)	724 (11.1)	2230 (8.8)
Diabetes					
Type 1	48 (0.4)	24 (0.5)	17 (0.9)	46 (0.7)	135 (0.5)
Type 2	771 (6.3)	457 (9.6)	306 (15.4)	709 (10.9)	2243 (8.8)
Neither	11,305 (92.9)	4262 (89.1)	1628 (81.9)	5680 (87.4)	22,875 (89.9)
Missing	49 (0.4)	40 (0.8)	38 (1.9)	64 (1.0)	191 (0.8)
Hypertension					
Yes	4279 (35.2)	2130 (44.5)	1192 (59.9)	3215 (49.5)	10,816 (42.5)
No	7662 (62.9)	2556 (53.4)	744 (37.4)	3076 (47.3)	14,038 (55.2)
Missing	232 (1.9)	97 (2.0)	53 (2.7)	208 (3.2)	590 (2.3)
Alcohol consumption					
Regular drinker	9691 (79.6)	3571 (74.7)	1210 (60.8)	4586 (70.6)	19,058 (74.9)
Occasional drinker	1176 (9.7)	597 (12.5)	347 (17.4)	934 (14.4)	3054 (12.0)
No drinking	1096 (9.0)	535 (11.2)	352 (17.7)	801 (12.3)	2784 (10.9)
Missing	210 (1.7)	80 (1.7)	80 (4.0)	178 (2.7)	548 (2.2)
Smoking (categorical)					
Nonsmoker	6265 (51.5)	2203 (46.1)	896 (45.0)	2850 (43.9)	12,214 (48.0)
Daily smoker	680 (5.6)	329 (6.9)	152 (7.6)	480 (7.4)	1641 (6.4)
Occasional smoker	182 (1.5)	93 (1.9)	29 (1.5)	90 (1.4)	394 (1.5)
Ex-smoker	4346 (35.7)	1868 (39.1)	809 (40.7)	2688 (41.4)	9711 (38.2)
Missing	700 (5.8)	290 (6.1)	103 (5.2)	391 (6.0)	1484 (5.8)
Smoking (cigarette-years)					
Mean (SD)	30.0 (52.6)	38.7 (61.4)	49.3 (73.7)	45.3 (69.4)	37.0 (61.2)
Median [Min, Max]	0 [0, 490]	0 [0, 490]	2.00 [0, 392]	3.00 [0, 490]	0 [0, 490]
Missing	799 (6.6)	333 (7.0)	117 (5.9)	456 (7.0)	1705 (6.7)
Whole-body bone mineral density					
Mean (SD)	1.12 (0.146)	1.14 (0.132)	1.14 (0.137)	1.12 (0.139)	1.14 (0.136)
Median [Min, Max]	1.11 [0.73, 1.84]	1.14 [0.71, 1.99]	1.14 [0.70, 1.78]	1.12 [0.72, 1.93]	1.13 [0.70, 1.99]
Missing	107 (5.4)	385 (3.2)	189 (4.0)	251 (3.9)	93 (2.7)

### Statistical Analysis

To answer our research question, we conducted the statistical analysis in 2 steps. First, we conducted latent class analysis (LCA) to identify unobserved oral health and cognitive health profiles within our sample. These unobserved categorical variables were identified based on the pattern of the observed/measured variables (i.e., indicators of oral health and cognitive health variables). Second, we used these categorical variables as the outcome variables and BMD as the main exposure variable in 2 series of multinomial logistic regression models.

We conducted all statistical analyses using R statistical software. The poLCA package was specifically used for the LCA. To address missing data, we used complete case analysis.

#### Step 1: LCA

Prior to running the cluster analysis, the Spearman correlation coefficient was used to detect collinearity between cognitive measures, and those variables with a high level of correlation (correlation coefficient of >0.6) were removed from the model. The high correlation between the REY trial I and REY trial II (*r* = 0.68) resulted in exclusion of the latter. This decision was based on the fact that an individual with impaired immediate recall has a higher chance of showing impaired performance on delayed recall as well. After running the correlation and removing highly correlated variables, 7 variables (i.e., REY trial I, Mental Alternation Test (MAT), Animal Fluency (AF) test, event-based and time-based prospective memory tasks, individual sub-scores of the COWAT, and the interference ratio from the Stroop test) were dichotomized using the median and included in the cluster analysis. For oral health indicators, we transformed responses from Likert scale to binary format, to improve the interpretability of oral health classes.

We estimated 2 to 7 class models and used the Bayesian information criterion (BIC), Akaike information criterion (AIC), and our theoretical understanding to identify the models with the best fit for both oral cognitive health. For each individual in the sample, LCA provides the probability of membership in each of the identified classes. Maximum inclusion probability was then used to assign each individual to only 1 class of the latent variables.

#### Step 2: Regression analysis

Using the oral health and cognitive health categories as the outcome variables, 2 series of multinomial logistic regressions were fitted. We used these models to estimate the extent to which cholinergic system activity, measured by BMD, can explain the oral health and cognitive health classifications after adjusting for different subsets of potential confounders. Analysis output was presented in terms of odds ratios and 95% confidence interval (CI).

## Results

### Oral and Cognitive Health Profiles

Two- to 7-class models were analyzed for oral health and 2- to 5-class models were tested for cognitive health. While the 6-class solution had the best model fit for oral health (AIC = 283,289.2, BIC = 284,470.8), the 5-class solution was selected for oral health cluster analysis, considering the clinical interpretability of the models (AIC = 298,952.5, BIC = 299,977.1). As for the cognitive health, the 4-class model was chosen since it presented the best model fit (AIC = 185,680.4 and BIC = 185,901.7) and good interpretability. After choosing the best model, individuals were assigned to latent classes based on their maximum class membership probability.

Figure A presents the heat map of the likelihood of reporting “favorable oral health” for individuals in each class. Based on the patterns of responses to different questions, oral health classes were described as class 1 (*n* = 15,922; 62.6%), the best oral health class; class 2 (*n* = 882; 3.2%), denture wearers with poor oral health; class 3 (*n* = 1320; 5.2%), non–denture wearers with poor oral health; class 4 (*n* = 2251; 8.8%), denture wearers with good oral health; and class 5 (*n* = 5129; 20.2%), non–denture wearers with moderate oral health status.

**Figure 1.**
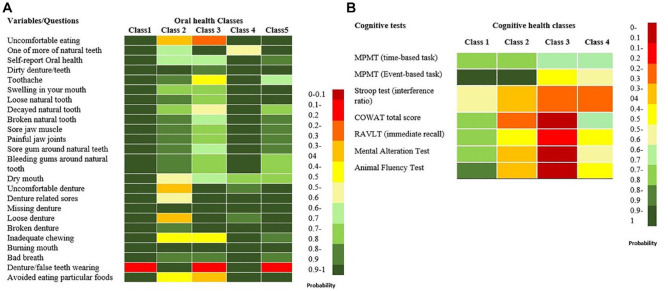
Heat map visualizing: (**A**) probability of individuals in each class to report favorable oral health, (**B**) likelihood of performing higher than median in cognitive test in each class.

For individuals in each cognitive health class, Figure B visualizes the probability of performing higher than median in each cognitive test. Based on this pattern, the cognitive health classes were defined as class 1 (*n* = 12,173; 47.8%), good cognitive performance; class 2 (*n* = 4754; 18.8%), poor verbal fluency; class 3 (*n* = 1989; 7.8%), poor cognitive performance (poor verbal fluency and memory); and class 4 (*n* = 6499; 25.5%), low memory performance. The descriptive statistics of basic sociodemographic variables and confounders by each cluster are presented in Tables 2 and 3.

### Regression Models

Six models were fitted to the data for each outcome variable, namely, oral health and cognitive health status. For the first set of regressions, oral health was the outcome variable and class 2 (the poorest oral health status) was considered as the reference (Table 4). In all models, a 1-unit increase in BMD was associated with higher odds of falling into other classes rather than class 2. After adjusting for all confounders (model 6), a 1-unit increase in the BMD score was associated with 1.58 (95% CI: 0.85–2.92) times odds of membership in best oral health class compared with odds of class 2 membership.

**Table 4.** Multinomial Regression Models for the Associations between Bone Mineral Density Scores and Oral/Cognitive Health Classes.

Oral Health	Model 1^a^	Model 2^b^	Model 3^c^	Model 4^d^	Model 5^e^	Model 6^f^
Oral health						
Denture poor oral health (class 2)	1	1	1	1	1	1
Nondenture poor oral health (class 3)	2.72 [1.29, 5.75]	1.35 [0.58, 3.13]	1.33 [0.57, 3.07]	1.23 [0.53, 2.81]	1.46 [0.67, 3.17]	1.31 [0.6, 2.83]
Denture good oral health (class 4)	1.38 [0.69, 2.76]	1.45 [0.68, 3.05]	1.34 [0.64, 2.82]	1.23 [0.59, 2.58]	1.38 [0.69, 2.75]	1.26 [0.63, 2.5]
Nondenture moderate oral health (class 5)	6.54 [3.46, 12.35]	2.5 [1.25, 5.05]	2.25 [1.11, 4.54]	1.81 [0.9, 3.66]	2.49 [1.3, 4.75]	1.94 [1.2, 3.7]
Best oral health (class 1)	5.81 [3.16, 10.66]	2.2 [1.13, 4.27]	1.84 [0.94, 3.6]	1.41 [0.72, 2.76]	2.16 [1.17, 3.99]	1.58 [0.85, 2.92]
Cognitive health						
Lowest cognitive performance (class 3)	1	1	1	1	1	1
Low verbal fluency (class 2)	3.03 [1.93, 4.76]	1.72 [1.05, 2.83]	1.62 [0.98, 2.86]	1.53 [0.92, 2.5]	1.71 [1.03, 2.84]	1.61 [0.97, 2.66]
Poor cognitive performance (class 4)	1.6 [1.03, 2.48]	1.2 [0.75, 1.91]	1.19 [0.74–1.91]	1.13 [0.7, 1.81]	1.26 [0.78, 2.02]	1.19 [0.74, 1.92]
Best cognitive performance (class 1)	4.32 [0.86, 6.5]	1.82 [1.14, 2.9]	1.58 [0.98, 2.55]	1.44 [0.89, 2.32]	1.79 [1.11, 2.88]	1.61 [1.00, 2.6]

Class 2 is the reference group.

a Crude association between bone mineral density score and oral health status classification.

b Estimation adjusted for age and sex.

c Estimation adjusted for age, sex, education, total household income, and ethnicity.

d Estimation adjusted for age, sex, education, total household income, ethnicity, smoking (categorical), smoking (cigarette-years).

e Estimation adjusted for age, sex, education, total household income, ethnicity, smoking (categorical), smoking (cigarette-years), hypertension, and diabetes.

f Estimation adjusted for age, sex, education, total household income, ethnicity, smoking (categorical), smoking (cigarette-years), alcohol consumption, diabetes, hypertension.

In the second set of regressions, cognitive health was considered as the outcome variable, with class 3 (the lowest cognitive function) being the reference group (Table 4). In all models, the odds of falling into class 4 were lower than the odds of falling into class 2 and 1, when BMD was increased for 1 unit. After adjusting for all confounders (model 6), a 1-unit increase in the BMD score was associated with 1.61 (95% CI: 1–2.6) times odds of membership in class 1 compared with odds of class 3 membership.

## Discussion

To date, an overwhelming number of studies have focused on the direction and magnitude of the association between oral and cognitive health (Wu et al. 2016). Yet, these studies investigating the causal relationship are inconsistent and fail to consider other underlying common contributors. To our knowledge, this study is the first to identify clusters of oral and cognitive health and to assess the decline of oral and cognitive health from a cholinergic perspective, which is conceptually different from the earlier proposed mechanisms and provides a better understanding of oral-systemic health association.

Regarding the oral health cluster characteristics, individuals in class 1 comprised most of the study sample, reporting the best oral health among all classes. This finding is in tune with a survey of a Canadian representative population, which reported that 84% of Canadians termed their oral health as excellent or good (Health Canada 2010). A moderate to poor oral health was observed among non–denture-wearing clusters (class 3 and 5). This suggests that the oral health problems in these classes stem from “natural teeth” rather than the prosthesis, as they had a low probability of reporting uncomfortable dentures or denture-related sores. Class 2 and class 4 clusters had the highest probability of wearing dentures and being edentulous. However, the class 4 cluster reported better oral health in comparison with a non–denture-wearing cluster such as class 3. These findings, in accordance with other studies, might suggest that adequate oral rehabilitation using complete dentures tends to improve patient-perceived oral health (Thalji et al. 2016).

Considering the overall picture, denture-wearing clusters (class 2 and 4) were older, had a lower educational level, and reported lower household income in comparison with non–denture-wearing clusters. In addition, these classes were characterized by a higher proportion of patients with chronic conditions, such as hypertension and diabetes mellitus type 2. These findings are in line with a study suggesting a strong association between tooth loss and 5-y age increase in elderly individuals (Thalji et al. 2016), lower education level, lower income, and tobacco consumption (Gilbert et al. 2003; Hanioka et al. 2011; Seerig et al. 2015).

Among cognitive health clusters, individuals in class 3 were the oldest, had the highest mean tobacco consumption, and had the highest proportion of chronic health conditions. This group also showed the lowest total household income and education level. Expectedly, this group fared poorly on the cognitive domain. In contrast, class 1 individuals with the best cognitive performance were younger, had the highest level of education, and had the highest total household income, with the majority being nonsmokers.

These findings are in accordance with the results of other studies reporting an association between cognitive function in the elderly and education attainment (Fletcher et al. 2021), income (Lee et al. 2010), hypertension (Forte et al. 2020), diabetes mellitus type 2 (Ahtiluoto et al. 2010), and smoking (Peters et al. 2008). In addition, the cumulative effect of exposure to lower socioeconomic status throughout an individual’s lifetime on cognitive function has been supported by different studies using life course approaches (Nguyen et al. 2008).

Class 4 was the second oldest group, which was composed of participants reporting lower education, inferior income level, and worse performance on retrospective and prospective memory tasks compared to classes 1 and 2. These findings could be explained by the differential effect of age on prospective and retrospective memory. This is in accordance with some studies reporting a higher impact of aging on prospective memory tasks as they require more self-initiation (McDaniel and Einstein 2000). Surprisingly, class 4 had a higher probability of performing higher than median in verbal fluency tasks compared with class 2. The clustering pattern might reflect only the moderate correlation between the memory and verbal fluency of adults (Kavé and Sapir-Yogev 2020), possibly because these functions are processed by different brain regions (Ghanavati et al. 2019).

It is interesting to note that a unit increase in BMD leads to a mild increase in the odds of being placed in a better oral health class, although this was not statistically significant. Based on this observation, estimation of cholinergic neurons’ activity via BMD could not explain the clustering of the participants based on their oral health status. However, as we adjusted for more potential covariates (moving from model 1 to model 6), the odds of class membership, especially for classes with better oral health, reduced (Table 4). This finding could be attributed to the influence of various confounding factors, which explain the variability in class memberships and not the BMD per se. This could be due to the fact that BMD is a complex parameter that hinges on a variety of factors such as medication (Ghosh and Majumdar 2014), genetics (Livshits et al. 2004), and diet (Denova-Gutiérrez et al. 2018). The results of this study perhaps indicate that cholinergic activity assessment is more than being solely dependent on BMD.

Similar to the results of regression models performed to explain oral health clustering, an increase in BMD was not associated with a higher odds of membership in any of the cognitive health classes compared with the reference group (i.e., the class with the worst cognitive health). Therefore, the use of BMD score as a measure of cholinergic neurons’ activity could not explain the clustering of the study sample based on their cognitive health status. Nevertheless, the point estimates of odds ratios for the group with poor memory performance (class 4) were the lowest in all models compared with the other groups (see Table 4).

Although the measurement of BMD as an indication of cholinergic neurons’ activity could not explain the oral and cognitive health class memberships, adjusting for socioeconomic status (SES) in both sets of regression models reduced the odds ratios, especially for the groups with better oral and cognitive health status. Although this study could not use a life course approach in the analysis, these findings may support the notion that any potential association between poor oral health and cognitive health might be explained by common explanatory factors such as exposure to lower SES, as has been recently mentioned by Thomson and Barak (2021).

One of the main limitations of this study is the cross-sectional nature of the analyses, which limits our capability to infer causality based on our findings. However, this study provides exploratory analyses and investigates a new hypothesis explaining the class membership via cholinergic activity. Another limitation of this study was the selection of BMD as the proxy measure for cholinergic neurons’ activity. Hence, the results of our analyses should be interpreted with caution. It is important to mention that taking cholinergic or anticholinergic medications could be a confounder variable in the association between BMD and oral and cognitive health. At the time of our analyses, data on medication were not available; hence we could not adjust for the effect of medication. Nevertheless, the exploratory nature of this study justifies the use of this measure based on the literature. It is recommended that confounder variables such as medications and other health conditions be taken into account in future studies. In addition, the use of self-reported oral health measures instead of clinical examination might limit our ability to accurately assess the oral health of the study participants. However, these self-reported oral health questionnaires have shown acceptable reliability and validity (Locker and Miller 1994).

Another limitation of this study that is worth mentioning is that, in our final regression models, we used a complete case analysis to handle missingness in the data. Because the present conclusions are conservative (e.g., multivariate regression analysis does not support rejection of H_0_), it is unlikely that imputation would affect conclusions. Also, Table 4 in the appendix presents the difference between the Comprehensive cohort of CLSA and our final sample with complete cases in terms of basic demographic factors. Finally, we did not explore the association between oral health and cognitive status in this study, and this can be studied in future research.

Despite these limitations, the large sample size was one of the main assets of the present study, which adds precision to the statistical analyses. This study controlled for a variety of covariates, including demographic characteristics, chronic conditions, and health-related behaviors. Moreover, LCA was used as a novel analysis to cluster a Canadian sample with heterogeneous oral health and cognitive health profiles. Analyses of cluster-specific patterns may be especially important for the design of future longitudinal studies, which can lead to more powerful assumptions regarding whether oral and cognitive health are associated with cholinergic neurons’ activity.

Further valid measures of cholinergic activity should be tested in future studies. In addition, the observations of this study suggest that the life course epidemiological concepts tackling environmental and socioeconomic factors might be more powerful determinants of oral and cognitive health.

## Conclusion

Within the limitation of this exploratory study, the results of the cluster analysis suggest that the deterioration of oral and cognitive health in this Canadian elderly cohort is not attributed to the age-related changes in cholinergic activity based on BMD as a proxy measure.

## Author Contributions

K. Rohani, contributed to conception and design, data acquisition, drafted the manuscript; B. Nicolau, L. Booij, contributed to conception and design, data interpretation, critically revised the manuscript; S. Madathil, contributed to conception and design, data acquisition and analysis, critically revised the manuscript; D. Jafarpour, P.B. Haricharan, contributed to data interpretation, drafted the manuscript; J. Feine, R. Alchini, F. Tamimi, contributed to data conception and design, critically revised the manuscript; R. de Souza, contributed to conception and design, data acquisition, analysis, and interpretation, critically revised the manuscript. All authors gave their final approval and agree to be accountable for all aspects of the work.

## Supplemental Material

sj-docx-1-jct-10.1177_23800844231190834 – Supplemental material for A Cluster Analysis of Oral and Cognitive Health Indicators: An Exploratory Study on Cholinergic Activity as the Link Supplemental material, sj-docx-1-jct-10.1177_23800844231190834 for A Cluster Analysis of Oral and Cognitive Health Indicators: An Exploratory Study on Cholinergic Activity as the Link by K. Rohani, B. Nicolau, S. Madathil, L. Booij, D. Jafarpour, P.B. Haricharan, J. Feine, R. Alchini, F. Tamimi and R. de Souza in JDR Clinical & Translational Research
