# Supplementary material for: A Cluster Analysis of Oral and Cognitive Health Indicators in the CLSA: An Exploratory Study on Cholinergic Activity as the Link
Source: JDR Clin Trans Res. 2023 Aug 22;9(3):294–305. doi: 10.1177/23800844231190834 (PMC11184914; doi:10.1177/23800844231190834)
Supplement: sj-docx-1-jct-10.1177_23800844231190834 – Supplemental material for A Cluster Analysis of Oral and Cognitive Health Indicators: An Exploratory Study on Cholinergic Activity as the Link [file sj-docx-1-jct-10.1177_23800844231190834.docx]

**Title: Investigation of a potential pathway influencing oral and cognitive health**

Authors: Kimia Rohani, Belinda Nicolau, Sreenath Madathil, Linda Booij, Dana Jafarpour, Praveen Bhoopathi Haricharan, Jocelyne Feine, Ricardo Alchini, Faleh Tamimi, Raphael de Souza

**Appendix Table 1.** Oral health questions from Maintaining Contact Questionnaire and their response categories included in the latent class analysis step.

|  | **Question (Q)** | **Response categories in the final dataset** |
| --- | --- | --- |
| **1** | In general, would you say the health of your mouth is excellent, very good, good, fair, or poor? | Excellent, Very Good, Good 1  Fair, Poor 2 |
| **2** | Do you have one or more of your own original teeth? | Yes 1  No 2 |
| 3 | Do you wear dentures or false teeth? | Yes 1  No 2 |
| 4 | In the past 12 months, how often have you found it uncomfortable to eat any food because of problems with your mouth? Would you say… | Often, Sometimes 2  Rarely, Never 1 |
| 5 | In the past 12 months, how often have you avoided eating particular foods because of problems with your mouth? Would you say… | Often, Sometimes 2  Rarely, Never 1 |
| 6 | In the past 12 months have you experienced any of the following? | |
|  | 6.1. Toothache | Yes 2, No 1 |
|  | 6.2. Cannot chew adequately | Yes 2, No 1 |
|  | 6.3. Dentures uncomfortable | Yes 2, No 1 |
|  | 6.4. Dentures loose/don’t fit | Yes 2, No 1 |
|  | 6.5. Dentures broken | Yes 2, No 1 |
|  | 6.6. Dentures missing | Yes 2, No 1 |
|  | 6.7. Swelling in your mouth | Yes 2, No 1 |
|  | 6.8. Dry mouth | Yes 2, No 1 |
|  | 6.9. Burning mouth | Yes 2, No 1 |
|  | 6.10. Jaw muscles sore | Yes 2, No 1 |
|  | 6.11. Jaw joints painful | Yes 2, No 1 |
|  | 6.12. Natural tooth decayed | Yes 2, No 1 |
|  | 6.13. Natural tooth loose | Yes 2, No 1 |
|  | 6.14. Natural tooth broken | Yes 2, No 1 |
|  | 6.15. Gums around natural teeth are sore | Yes 2, No 1 |
|  | 6.16. Gums around natural teeth bleed | Yes 2, No 1 |
|  | 6.17. Denture-related sores | Yes 2, No 1 |
|  | 6.18. Teeth or dentures dirty | Yes 2, No 1 |
|  | 6.19. Bad breath | Yes 2, No 1 |

**Appendix Table 2.** Socio-demographic characteristics of the CLSA comprehensive cohort

|  | **Overall N=30,097(%)** |
| --- | --- |
| **Age(years)** |  |
| Mean (SD) | 63.0 (10.3) |
| Median [Min, Max] | 62.0 [45.0, 86.0] |
| **Sex** |  |
| Male | 15320 (50.9) |
| Female | 14777 (49.1) |
| **Education** |  |
| Less than secondary | 1643 (5.5) |
| Secondary | 2839 (9.4) |
| Some post-secondary | 2238 (7.4) |
| Post-secondary degree | 23327 (77.5) |
| Missing | 50 (0.2) |
| **Total household income** |  |
| < $20,000 | 1566 (5.2) |
| ≥ $20,000 and < $50,000 | 6360 (21.1) |
| ≥ $50,000 and< $100,000 | 9907 (32.9) |
| ≥ $100,000 and< $150,000 | 5524 (18.4) |
| ≥ $150,000 | 4799 (15.9) |
| Missing | 1941 (6.4) |
| **Ethnicity** |  |
| White | 27412 (91.1) |
| Non-white | 1303 (4.3) |
| Missing | 1382 (4.6) |

**Appendix Table 3.** Descriptive statistics of oral health variables by age group in the CLSA comprehensive cohort

|  | **45-54 N=7,595 (%)** | **55-64 N=9,856 (%)** | **65-74 N=7,362(%)** | **+75 N=5,284(%)** | **Overall N=30,097(%)** |
| --- | --- | --- | --- | --- | --- |
| **Self-rated oral health** |  |  |  |  |  |
| Excellent | 2590 (34.1) | 3098 (31.4) | 2272 (30.9) | 1423 (26.9) | 9383 (31.2) |
| Very good | 2846 (37.5) | 3815 (38.7) | 2795 (38.0) | 1918 (36.3) | 11374 (37.8) |
| Good | 1389 (18.3) | 1942 (19.7) | 1558 (21.2) | 1290 (24.4) | 6179 (20.5) |
| Fair | 325 (4.3) | 484 (4.9) | 359 (4.9) | 247 (4.7) | 1415 (4.7) |
| Poor | 82 (1.1) | 144 (1.5) | 96 (1.3) | 65 (1.2) | 387 (1.3) |
| Missing | 363 (4.8) | 373 (3.8) | 282 (3.8) | 341 (6.5) | 1359 (4.5) |
| **Having one or more of your original teeth** | | | | | |
| Yes | 7114 (93.7) | 9125 (92.6) | 6456 (87.7) | 4206 (79.6) | 26901 (89.4) |
| No | 125 (1.6) | 368 (3.7) | 638 (8.7) | 755 (14.3) | 1886 (6.3) |
| Missing | 356 (4.7) | 363 (3.7) | 268 (3.6) | 323 (6.1) | 1310 (4.4) |
| **Denture wearing** |  |  |  |  |  |
| Yes | 668 (8.8) | 1866 (18.9) | 2401 (32.6) | 2338 (44.2) | 7273 (24.2) |
| No | 6570 (86.5) | 7624 (77.4) | 4692 (63.7) | 2623 (49.6) | 21509 (71.5) |
| Missing | 357 (4.7) | 366 (3.7) | 269 (3.7) | 323 (6.1) | 1315 (4.4) |
| **Uncomfortable eating** |  |  |  |  |  |
| Often | 157 (2.1) | 224 (2.3) | 184 (2.5) | 124 (2.3) | 689 (2.3) |
| Sometimes | 562 (7.4) | 766 (7.8) | 522 (7.1) | 412 (7.8) | 2262 (7.5) |
| Rarely | 1573 (20.7) | 1943 (19.7) | 1348 (18.3) | 891 (16.9) | 5755 (19.1) |
| Never | 4937 (65.0) | 6548 (66.4) | 5029 (68.3) | 3526 (66.7) | 20040 (66.6) |
| Missing | 366 (4.8) | 375 (3.8) | 279 (3.8) | 331 (6.3) | 1351 (4.5) |
| **Eating avoidance due to Oral problems** | | | | | |
| Often | 101 (1.3) | 175 (1.8) | 145 (2.0) | 107 (2.0) | 528 (1.8) |
| Sometimes | 291 (3.8) | 461 (4.7) | 378 (5.1) | 308 (5.8) | 1438 (4.8) |
| Rarely | 809 (10.7) | 1077 (10.9) | 758 (10.3) | 552 (10.4) | 3196 (10.6) |
| Never | 6026 (79.3) | 7772 (78.9) | 5806 (78.9) | 3980 (75.3) | 23584 (78.4) |
| Missing | 368 (4.8) | 371 (3.8) | 275 (3.7) | 337 (6.4) | 1351 (4.5) |
| **Toothache** |  |  |  |  |  |
| Yes | 1139 (15.0) | 1309 (13.3) | 784 (10.6) | 422 (8.0) | 3654 (12.1) |
| No | 6100 (80.3) | 8185 (83.0) | 6310 (85.7) | 4540 (85.9) | 25135 (83.5) |
| Missing | 356 (4.7) | 362 (3.7) | 268 (3.6) | 322 (6.1) | 1308 (4.3) |
| **Chewing inadequacy** |  |  |  |  |  |
| Yes | 557 (7.3) | 791 (8.0) | 619 (8.4) | 458 (8.7) | 2425 (8.1) |
| No | 6682 (88.0) | 8703 (88.3) | 6475 (88.0) | 4504 (85.2) | 26364 (87.6) |
| Missing | 356 (4.7) | 362 (3.7) | 268 (3.6) | 322 (6.1) | 1308 (4.3) |
| **Uncomfortable denture** |  |  |  |  |  |
| Yes | 87 (1.1) | 253 (2.6) | 357 (4.8) | 329 (6.2) | 1026 (3.4) |
| No | 7152 (94.2) | 9241 (93.8) | 6737 (91.5) | 4633 (87.7) | 27763 (92.2) |
| Missing | 356 (4.7) | 362 (3.7) | 268 (3.6) | 322 (6.1) | 1308 (4.3) |
| **Loose denture** |  |  |  |  |  |
| Yes | 78 (1.0) | 260 (2.6) | 367 (5.0) | 381 (7.2) | 1086 (3.6) |
| No | 7161 (94.3) | 9234 (93.7) | 6727 (91.4) | 4581 (86.7) | 27703 (92.0) |
| Missing | 356 (4.7) | 362 (3.7) | 268 (3.6) | 322 (6.1) | 1308 (4.3) |
| **Broken denture** |  |  |  |  |  |
| Yes | 28 (0.4) | 97 (1.0) | 90 (1.2) | 81 (1.5) | 296 (1.0) |
| No | 7211 (94.9) | 9397 (95.3) | 7004 (95.1) | 4881 (92.4) | 28493 (94.7) |
| Missing | 356 (4.7) | 362 (3.7) | 268 (3.6) | 322 (6.1) | 1308 (4.3) |
| **Missing denture** |  |  |  |  |  |
| Yes | 8 (0.1) | 14 (0.1) | 23 (0.3) | 18 (0.3) | 63 (0.2) |
| No | 7231 (95.2) | 9480 (96.2) | 7071 (96.0) | 4944 (93.6) | 28726 (95.4) |
| Missing | 356 (4.7) | 362 (3.7) | 268 (3.6) | 322 (6.1) | 1308 (4.3) |
| **Swelling in mouth** |  |  |  |  |  |
| Yes | 363 (4.8) | 481 (4.9) | 332 (4.5) | 196 (3.7) | 1372 (4.6) |
| No | 6876 (90.5) | 9013 (91.4) | 6762 (91.9) | 4766 (90.2) | 27417 (91.1) |
| Missing | 356 (4.7) | 362 (3.7) | 268 (3.6) | 322 (6.1) | 1308 (4.3) |
| **Dry mouth** |  |  |  |  |  |
| Yes | 809 (10.7) | 1545 (15.7) | 1550 (21.1) | 1357 (25.7) | 5261 (17.5) |
| No | 6430 (84.7) | 7949 (80.7) | 5544 (75.3) | 3605 (68.2) | 23528 (78.2) |
| Missing | 356 (4.7) | 362 (3.7) | 268 (3.6) | 322 (6.1) | 1308 (4.3) |
| **Burning mouth** |  |  |  |  |  |
| Yes | 89 (1.2) | 142 (1.4) | 135 (1.8) | 77 (1.5) | 443 (1.5) |
| No | 7150 (94.1) | 9352 (94.9) | 6959 (94.5) | 4885 (92.4) | 28346 (94.2) |
| Missing | 356 (4.7) | 362 (3.7) | 268 (3.6) | 322 (6.1) | 1308 (4.3) |
| **Sore jaw muscle** |  |  |  |  |  |
| Yes | 570 (7.5) | 562 (5.7) | 295 (4.0) | 173 (3.3) | 1600 (5.3) |
| No | 6669 (87.8) | 8932 (90.6) | 6799 (92.4) | 4789 (90.6) | 27189 (90.3) |
| Missing | 356 (4.7) | 362 (3.7) | 268 (3.6) | 322 (6.1) | 1308 (4.3) |
| **Jaw joint pain** |  |  |  |  |  |
| Yes | 562 (7.4) | 609 (6.2) | 347 (4.7) | 206 (3.9) | 1724 (5.7) |
| No | 6677 (87.9) | 8885 (90.1) | 6747 (91.6) | 4756 (90.0) | 27065 (89.9) |
| Missing | 356 (4.7) | 362 (3.7) | 268 (3.6) | 322 (6.1) | 1308 (4.3) |
| **Decayed natural tooth** | | | | | |
| Yes | 994 (13.1) | 1419 (14.4) | 1102 (15.0) | 686 (13.0) | 4201 (14.0) |
| No | 6245 (82.2) | 8075 (81.9) | 5992 (81.4) | 4276 (80.9) | 24588 (81.7) |
| Missing | 356 (4.7) | 362 (3.7) | 268 (3.6) | 322 (6.1) | 1308 (4.3) |
| **Loose natural tooth** |  |  |  |  |  |
| Yes | 337 (4.4) | 516 (5.2) | 357 (4.8) | 177 (3.3) | 1387 (4.6) |
| No | 6902 (90.9) | 8978 (91.1) | 6737 (91.5) | 4785 (90.6) | 27402 (91.0) |
| Missing | 356 (4.7) | 362 (3.7) | 268 (3.6) | 322 (6.1) | 1308 (4.3) |
| **Broken natural tooth** |  |  |  |  |  |
| Yes | 779 (10.3) | 1123 (11.4) | 768 (10.4) | 484 (9.2) | 3154 (10.5) |
| No | 6460 (85.1) | 8371 (84.9) | 6326 (85.9) | 4478 (84.7) | 25635 (85.2) |
| Missing | 356 (4.7) | 362 (3.7) | 268 (3.6) | 322 (6.1) | 1308 (4.3) |
| **Soreness of the gums around natural teeth** | | | | | |
| Yes | 748 (9.8) | 864 (8.8) | 496 (6.7) | 226 (4.3) | 2334 (7.8) |
| No | 6491 (85.5) | 8630 (87.6) | 6598 (89.6) | 4736 (89.6) | 26455 (87.9) |
| Missing | 356 (4.7) | 362 (3.7) | 268 (3.6) | 322 (6.1) | 1308 (4.3) |
| **bleeding of gums around natural teeth** | | | | | |
| Yes | 1102 (14.5) | 1226 (12.4) | 638 (8.7) | 263 (5.0) | 3229 (10.7) |
| No | 6137 (80.8) | 8268 (83.9) | 6456 (87.7) | 4699 (88.9) | 25560 (84.9) |
| Missing | 356 (4.7) | 362 (3.7) | 268 (3.6) | 322 (6.1) | 1308 (4.3) |
| **Denture related sores** |  |  |  |  |  |
| yes | 66 (0.9) | 164 (1.7) | 244 (3.3) | 193 (3.7) | 667 (2.2) |
| no | 7173 (94.4) | 9330 (94.7) | 6850 (93.0) | 4769 (90.3) | 28122 (93.4) |
| Missing | 356 (4.7) | 362 (3.7) | 268 (3.6) | 322 (6.1) | 1308 (4.3) |
| **Dirty denture or teeth** |  |  |  |  |  |
| Yes | 266 (3.5) | 312 (3.2) | 165 (2.2) | 87 (1.6) | 830 (2.8) |
| No | 6973 (91.8) | 9182 (93.2) | 6929 (94.1) | 4875 (92.3) | 27959 (92.9) |
| Missing | 356 (4.7) | 362 (3.7) | 268 (3.6) | 322 (6.1) | 1308 (4.3) |
| **Bad breath** |  |  |  |  |  |
| Yes | 672 (8.8) | 724 (7.3) | 455 (6.2) | 226 (4.3) | 2077 (6.9) |
| No | 6567 (86.5) | 8770 (89.0) | 6639 (90.2) | 4736 (89.6) | 26712 (88.8) |
| Missing | 356 (4.7) | 362 (3.7) | 268 (3.6) | 322 (6.1) | 1308 (4.3) |

**Appendix Table 4**. Descriptive statistics of cognitive tests by age group in the CLSA comprehensive cohort

|  | **45-54 (N=7,595)** | **55-64 (N=9,856)** | **65-74 (N=7,362)** | **+75 (N=5,284)** | **Overall (N=30,097)** |
| --- | --- | --- | --- | --- | --- |
| **Rey Auditory Verbal Learning Test (RAVLT)-immediate recall** | | | | | |
| Mean (SD) | 6.51 (1.85) | 6.16 (1.82) | 5.59 (1.78) | 4.67 (1.70) | 5.85 (1.91) |
| Median [Min, Max] | 6.00 [0, 14.0] | 6.00 [0, 14.0] | 6.00 [0, 14.0] | 5.00 [0, 13.0] | 6.00 [0, 14.0] |
| Missing | 214 (2.8) | 297 (3.0) | 260 (3.5) | 249 (4.7) | 1020 (3.4) |
| **Mental Alteration Test (MAT)** | | | | | |
| Mean (SD) | 28.9 (8.58) | 27.6 (8.32) | 25.5 (8.44) | 22.4 (8.58) | 26.5 (8.75) |
| Median [Min, Max] | 30.0 [0, 51.0] | 28.0 [0, 51.0] | 26.0 [0, 51.0] | 22.0 [0, 51.0] | 27.0 [0, 51.0] |
| Missing | 275 (3.6) | 411 (4.2) | 400 (5.4) | 400 (7.6) | 1486 (4.9) |
| **Animal Fluency Test (AFT)** | | | | | |
| Mean (SD) | 23.8 (6.45) | 22.5 (6.22) | 20.2 (5.97) | 17.6 (5.50) | 21.4 (6.47) |
| Median [Min, Max] | 24.0 [0, 52.0] | 22.0[1.00,48.0] | 20.0 [0, 48.0] | 17.0 [0, 43.0] | 21.0 [0, 52.0] |
| Missing | 162 (2.1) | 208 (2.1) | 192 (2.6) | 169 (3.2) | 731 (2.4) |
| **Event-based task-Miami Prospective Memory Test (MPMT)** | | | | | |
| Mean (SD) | 8.76 (0.914) | 8.65 (1.10) | 8.35 (1.49) | 7.71 (1.98) | 8.44 (1.40) |
| Median [Min, Max] | 9.00 [0, 9.00] | 9.00 [0, 9.00] | 9.00 [0, 9.00] | 9.00 [0, 9.00] | 9.00 [0, 9.00] |
| Missing | 37 (0.5) | 79 (0.8) | 69 (0.9) | 59 (1.1) | 244 (0.8) |
| **Time-based task-Miami Prospective Memory Test (MPMT)** | | | | | |
| Mean (SD) | 8.82 (0.66) | 8.76 (0.75) | 8.63 (1.00) | 8.27 (1.44) | 8.66 (0.97) |
| Median [Min, Max] | 9.00 [0, 9.00] | 9.00 [0, 9.00] | 9.00 [0, 9.00] | 9.00 [0, 9.00] | 9.00 [0, 9.00] |
| Missing | 95 (1.3) | 133 (1.3) | 139 (1.9) | 123 (2.3) | 490 (1.6) |
| **Controlled Oral Word Association Test (COWAT)** | | | | | |
| Mean (SD) | 41.5 (12.4) | 40.3 (12.6) | 37.9 (12.7) | 35.6 (12.8) | 39.2 (12.8) |
| Median [Min, Max] | 41.0 [6.00, 99.0] | 40.0 [3.00, 105] | 37.0 [5.00, 86.0] | 35.0 [4.00, 90.0] | 39.0 [3.00, 105] |
| Missing | 201 (2.6) | 358 (3.6) | 291 (4.0) | 217 (4.1) | 1067 (3.5) |
| **Stroop test-interference ratio** | | | | | |
| Mean (SD) | 1.95 (0.54) | 2.10 (0.73) | 2.27 (0.79) | 2.42 (0.75) | 2.16 (0.73) |
| Median [Min, Max] | 1.88 [0.05, 19.5] | 2.00[0.06, 32.0] | 2.15 [0.04, 38.1] | 2.30 [0.09, 11.3] | 2.00 [0.045, 38.1] |
| Missing | 74 (1.0) | 125 (1.3) | 120 (1.6) | 102 (1.9) | 1. .4) |

**Appendix Table 5**. Comparison of the basic demographics of the Comprehensive cohort in CLSA with our study sample

|  | **Final Sample (N=25,444)** | **Comprehensive Cohort**  **(N=30,097)** |
| --- | --- | --- |
| **Age(years)** |  |  |
| Mean (SD) | 62.6 (10.1) | 63.0 (10.3) |
| Median [Min, Max] | 62.0 [45.0, 86.0] | 62.0 [45.0, 86.0] |
| **Sex** |  |  |
| male | 13035 (51.2%) | 15320 (50.9%) |
| female | 12409 (48.8%) | 14777 (49.1%) |
| **Education** |  |  |
| less than secondary | 1276 (5.0%) | 1643 (5.5%) |
| secondary | 2324 (9.1%) | 2839 (9.4%) |
| some post-secondary | 1844 (7.2%) | 2238 (7.4%) |
| post-secondary degree | 19963 (78.5%) | 23327 (77.5%) |
| Missing | 37 (0.1%) | 50 (0.2%) |
| **Total household income** |  |  |
| < $20,000 | 1202 (4.7%) | 1566 (5.2%) |
| ≥ $20,000 and < $50,000 | 5213 (20.5%) | 6360 (21.1%) |
| ≥ $50,000 and< $100,000 | 8503 (33.4%) | 9907 (32.9%) |
| ≥ $100,000 and< $150,000 | 4809 (18.9%) | 5524 (18.4%) |
| ≥ $150,000 | 4230 (16.6%) | 4799 (15.9%) |
| Missing | 1487 (5.8%) | 1941 (6.4%) |
| **Ethnicity** |  |  |
| white | 23214 (91.2%) | 27412 (91.1%) |
| non-white | 2230 (8.8%) | 1303 (4.3%) |
|  | | |


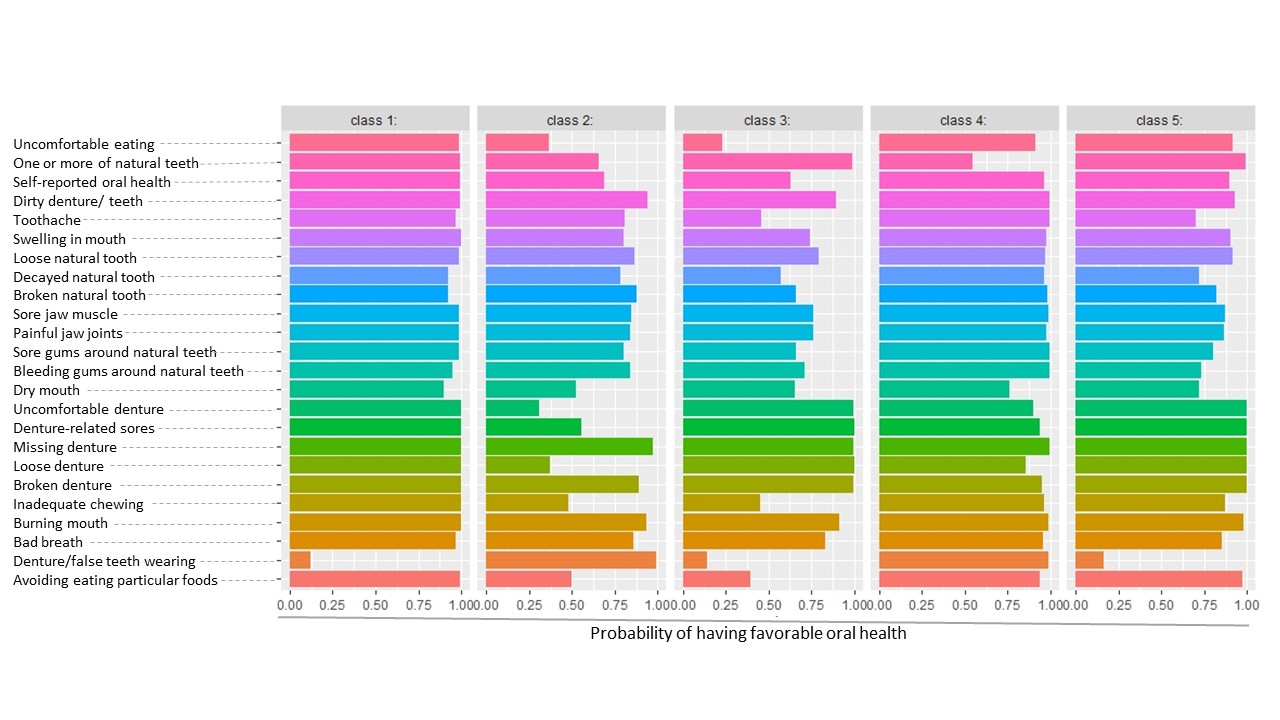


**Appendix Figure 1**. Bar graphs presenting probability of reporting favorable oral health for each variable in different oral health classes.


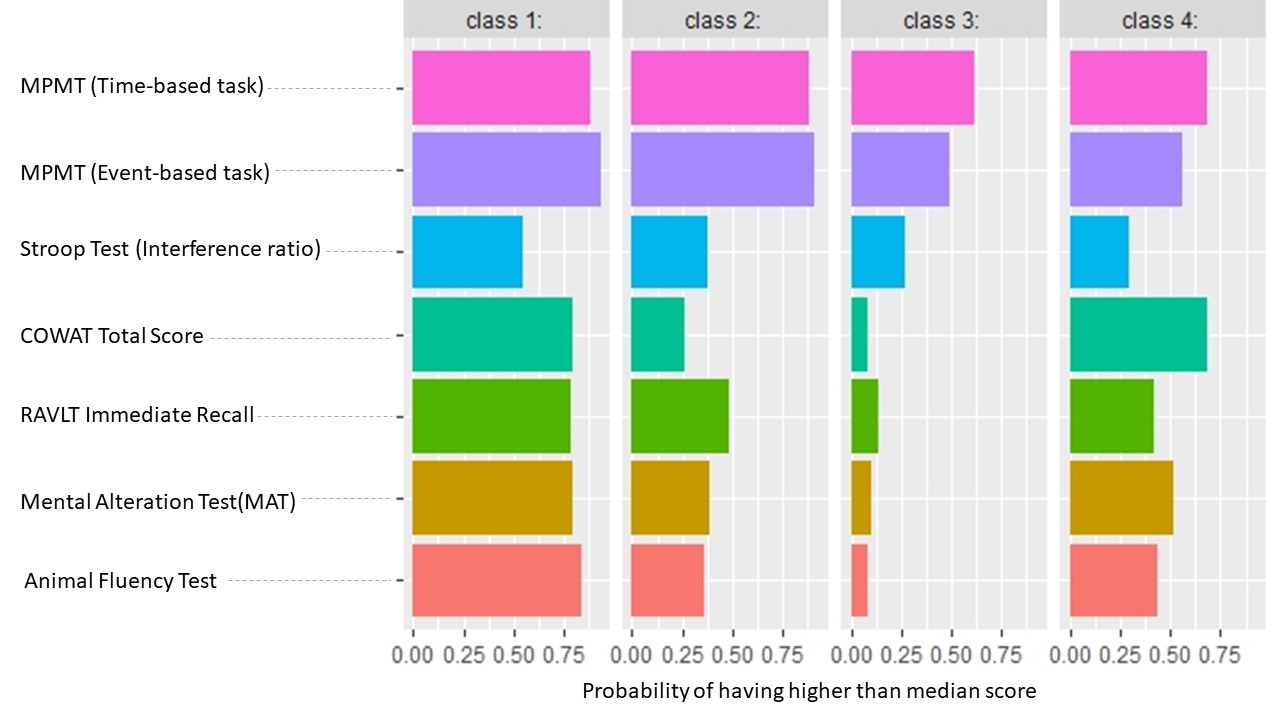


**Appendix Figure 2.** Bar graphs presenting probability of performing higher than median score for each cognitive test in different cognitive health classes.
